# “Provided a window on the world and lessened my feeling of isolation”: older adults’ perceived COVID-19 impact and technology use in Australia during recurrent lockdowns

**DOI:** 10.1186/s12877-024-04807-7

**Published:** 2024-02-28

**Authors:** Joyce Siette, Kristiana Ludlow, Laura Dodds, Paul Strutt, Viviana Wuthrich

**Affiliations:** 1 The MARCS Centre for Brain, Behaviour and Development, Western Sydney University, New South Wales, 2145 Australia; 2 Centre for Health Systems and Safety Research, Australian Institute of Health Innovation, Macquarie University, New South Wales, 2109 Australia; 3 Centre for Health Services Research, the University of Queensland, Queensland, 4102 Australia; 4 Lifespan Health Wellbeing Research Centre, Macquarie University, New South Wales, 2109 Australia

**Keywords:** COVID-19, Social isolation, Technology adoption, Older adults, Mental health

## Abstract

**Background:**

An informed understanding of older adults’ perceptions of the impact (positive or negative) of recurrent COVID-19 long lockdowns is important for the development of targeted interventions and resources for future restrictions. This study aimed to understand self-reported impacts of COVID-19 recurrent restrictions on older adults and how technology has been used to mitigate these.

**Methods:**

A cross-sectional national study of 257 community-dwelling older Australians based in Victoria (mean age = 67.6 years [SD = 7.2]; 20.6% male) completed an online or postal survey as part of a larger study examining the physical and mental health impacts of a second extended COVID-19 lockdown period. This secondary analysis reports on the findings from free-text responses to two open-ended questions included in that survey that asked participants to comment on the greatest impacts of the COVID-19 lockdowns (positive or negative) and the role of technology in supporting their wellbeing during this time. Responses were collected between July and September 2020. Data were analysed using content (COVID-19 impacts) and thematic (role of technology) analysis.

**Results:**

Respondents gave more negative responses (75.5%) than mixed (15.2%) and positive responses (6.2%) in reporting on the biggest impact of COVID-19 lockdowns. Inductive content analysis revealed two first-order main categories (Positive impacts and Negative impacts). Axial coding of main categories showed five second-order categories (Environmental, Physical Health, Social, Mental Health, and Personal) for both negative and positive main categories (totalling 10 second-order categories). Overall, respondents highlighted social loss as the key negative experience (70%), with acute feelings of social isolation contributing to negative impacts on mental wellbeing. The most commonly reported positive impact reported (11%) was having more time for relationships, relaxation, and new hobbies. Technology was primarily used to sustain socialisation and provide access to essential resources, services, and goods, which respondents perceived to contribute to maintaining their wellbeing.

**Conclusions:**

Findings suggest a critical need for interventions that address the social loss experienced by older adults during COVID-19 recurrent lockdowns, particularly to alleviate the associated negative impact on mental wellbeing. Recognising the positive aspect of increased time for relationships and leisure activities indicates potential areas for resilience-building strategies. The pivotal role of technology in mitigating adverse effects highlights its significance in building social connections and supporting overall wellbeing during challenging times. These implications can guide future efforts to enhance older adults’ resilience, mental health, and holistic wellbeing in future public health crises.

## Introduction

In response to the management of the COVID-19 pandemic, various countries utilised periods of lockdowns which greatly restricted people’s movements and time spent with other people outside of their physical residence [1–4]. In some areas, these lockdowns were recurrent and for extended durations [5]. The negative impacts of these long recurrent lockdowns on mental health and wellbeing have now been well established in the general population [6, 7]; however, it is less clear what older adults perceived to be the greatest impacts (negative or positive) and in what ways.

Contextually, from July to October 2020, individuals living in Victoria, Australia’s second largest State, underwent a lengthy (> 110 days) and severe lockdown to suppress the second wave of COVID-19 infections. This recurrent restriction started as Stage 3 “Stay-At-Home” orders [8–10] but later limited residents to essential travel confined to a 5 km radius, 1 h of daily outdoor exercise, an 8 pm to 5am curfew, and the closure of all non-essential businesses including retail stores, gyms, and hospitality sites [10, 11]. In response to Victoria’s COVID-19 crisis, other Australian State and Territory Governments effectively established constraints on interstate and border travel to minimise risks and contain the spread of the virus on a national scale [11]. Although lockdowns have been found to effectively decrease the spread of the virus [8], emerging research reinforces the link between reduced social interaction and poor psychological health as a consequence of infection-control interventions [12–14]. Detailed exploration of the perceived impact of persistent restrictions on older populations will provide a much needed understanding of these restrictions and contribute to identifying strategies that can mitigate the impact.

The effect of social isolation on older adults as a result of the COVID-19 pandemic has become an important issue of discussion and research [15]. A qualitative study of community-dwelling adults highlighted that a major repercussion of COVID-19 outbreaks was restrictions on socialisation [16]. A lack of social interaction has been shown to influence how people behave and interact with one another, and results in greater psychological distress, anxiety, anger and irritability [17, 18]. Challenges faced by older adults during the COVID-19 pandemic are similar, with studies highlighting persistent loneliness, poor emotional coping, and additionally, discomfort with new technologies as a means of staying connected with others [19]. Given these studies were conducted during the early phase of the pandemic using small sample sizes (27–151 individuals), more work needs to be undertaken in order to establish how older adults perceived and coped with lockdowns in order to inform management strategies in further outbreaks.

While there is some evidence that older adults utilised technology to support wellbeing during this period (e.g., [14, 20]), it is less clear what aspects of technology use aided wellbeing. During the COVID-19 pandemic, technology has become a critical avenue for supporting social connectivity. Evidence from early in the pandemic suggested a high proportion of older adults used technology for the first time to connect with others [14]. Chen et al.’s (2020) qualitative analysis of online discussion content amongst older adults during early stages of COVID-19 further indicated that online communication platforms helped them to stay connected with their family and the community [20]. This ability to stay connected to the world, not only through maintaining social connections but also having access to necessities through online shopping, was identified as crucial [20]. Older adults have also reported relationships with family and friends, the presence of digital social contact, and starting and/or maintaining hobbies, as sources of joy and comfort during the pandemic [21]. Using technology to sustain social contact has become an important mechanism for many older adults to cope during the pandemic as demonstrated in the US and UK [22]. Indeed, while previous studies have explored technology adoption during the initial phases of the COVID-19 pandemic [14, 23, 24], a critical gap remains in understanding how older adults' technology use evolves and adapts during subsequent lockdowns. The dynamic nature of the pandemic, coupled with potential changes in technology accessibility, societal responses, and individual experiences, highlights the importance of investigating technology use during a second lockdown. This study thus seeks to contribute to the existing knowledge by understanding older adults' technology behaviours in an evolving and iterative lockdown scenario, providing additional insights for policymakers, healthcare professionals, and researchers.

The present paper is part of a series of reports on the findings of a national survey on COVID-19 impact, quality of life, social networks, healthcare utilisation and technology use [25]. Quantitative results exploring impacts of the pandemic during restrictions associated with second lockdowns highlighted that COVID-19 had an impact, with 42.3% of older adults reporting that the pandemic had both positive and negative impacts, and 35.6% reported a negative impact only [25]. However, previous research highlights that quantitative data is of limited use in designing public health improvements as they lack in-depth description of the issues which matter to the targeted population [26]. An in-depth understanding of the positive and negative impacts of the pandemic from the perspective of older adults is thus needed.

While our prior larger cross-sectional national study aimed to explore state/territory differences of older adults' experiences during the pandemic, our present investigation concentrates specifically on the qualitative dimensions. The aim of this study was to therefore explore self-reported multifaceted impacts of recurrent COVID-19 restrictions on older adults, examining the role of technology in addressing and adapting to these impacts.

## Methods

### Study design and setting

A national cross-sectional survey was administered across Australia between 10th July and 28th September 2020, to overlap with the second lockdowns in the State of Victoria, which occurred between 8th July – 27th October 2020. The survey included 45 questions asking participants to reflect on the past four weeks on their experiences of the COVID-19 restrictions, including use of technology, access to healthcare services, and two-open ended questions about: a) self-perceived impacts of second COVID-19 restrictions; and b) how technology was adopted to support wellbeing. This study reports on the participants’ responses to the two open-ended questions. For further information about the other components of the survey (impact of COVID-19 lockdowns on quality of life, social networks and healthcare access), please refer to Siette et al. 2021 [25].

Including open-ended questions in surveys has the potential to provide comprehensive, contextual information and to give the public a voice to influence public health messaging as well as targeted improvement areas for health care and governmental systems [27, 28]. Furthermore, data from free-text responses often permit individuals to further describe their experiences in detail and offer an opportunity for members of the public to appraise and suggest improvements [29]. The recognition of the potential inherent in free-text responses, especially in national survey studies, has been steadily increasing due to their utility and narrative possibilities. Examining free-text data collected during the pandemic peak will also allow researchers and public health systems to investigate and respond to the evolving ways in which participants shape meaning beyond the scope of survey evaluation purposes [30–32].

### Participants and recruitment

Information about the survey and the opportunity to participate in the study was distributed throughout multiple channels including social media, e-newsletters, local council newspapers and flyers. The survey was delivered either online or via post to adults (*n* = 2,990) aged 55 years or more (M = 67.3, SD = 7.0, Range = 56–107) who were currently residing in Australia and had no self-reported diagnosis of dementia or other neurological disorders. A broad age range was included to increase the opportunity for sampling of Indigenous populations who are able to access aged care service from age 50 years [33]. The current analyses used data from the 257 respondents who resided in Victoria, a state experiencing the longest lockdown period at the time (M_age_ = 67.3, SD = 7.2). Further information of this group’s sociodemographics are available at Table 1 and Siette et al. 2021 [25]. Of these, 250 (97.3%) provided free-text responses regarding the impact of COVID-19 and 180 (70.0%) provided responses on how technology supported their wellbeing.

**Table 1:** Sample demographic characteristics based on the responses to two open-ended questions

Variable	Entire sample (*N* = 257) N (%)	COVID-19 impact (*N* = 191) N (%)	Technology (*N* = 242) N (%)
**Gender**			
Female	204 (79.4)	149 (78.4)	193 (79.8)
Male	53 (20.6)	41 (21.6)	49 (20.2)
**Age**			
**Mean [SD]**	67.6 [7.2]	67.8 [7.3]	67.5 [7.1]
55–64	103 (40.1)	59 (33.9)	79 (35.3)
65–74	116 (45.1)	85 (48.9)	111 (49.6)
75–84	35 (13.6)	27 (15.5)	31 (13.8)
85 +	3 (1.2)	3 (1.7)	3 (1.3)
**Socioeconomic status**			
1 (Lowest)	11 (4.3)	10 (5.3)	11 (4.8)
2	52 (20.2)	38 (20.0)	45 (19.8)
3	57 (22.2)	41 (21.6)	49 (21.6)
4	68 (26.5)	54 (28.4)	59 (26.0)
5 (Highest)	69 (26.8)	47 (24.7)	63 (27.8)
**Marital status**			
Never married	185 (6.8)	12 (7.1)	15 (6.9)
Married/De facto	1,755 (64.2)	101 (59.8)	131 (60.6)
Divorced/Separated but not divorced	476 (17.4)	37 (21.9)	45 (20.8)
Widowed	256 (9.4)	17 (10.1)	22 (10.2)
Unknown	61 (2.2)	2 (1.2)	3 (1.3)
**Country of birth**			
English-speaking country	204 (79.4)	154 (80.6)	196 (81.0)
Non-English-speaking country	53 (20.6)	37 (19.4)	46 (19.0)
**Educational attainment**			
Secondary School or less	41 (16.0)	30 (15.8)	38 (15.7)
Trade qualification	5 (2.0)	4 (2.1)	5 (2.1)
Certificate	21 (8.2)	15 (7.9)	19 (7.9)
Diploma	59 (23.0)	42 (22.1)	56 (23.1)
Bachelor’s Degree	55 (21.4)	39 (20.5)	51 (21.1)
Post-graduate degree	74 (28.8)	59 (31.1)	72 (29.8)
Unknown	2 (0.8)	1 (1.0)	1 (0.4)

### Ethics approval

All participants provided informed consent prior to completion of the survey. This study was approved by Macquarie University’s Human Research Ethics Committee (ref 6712).

### Measures

As part of the larger survey, two open-ended questions were asked “What has been the greatest impact of COVID-19 for you? This can be a positive or negative impact”, and “How have you used technology during COVID-19 to support your own wellbeing?” Demographic and health information including gender, age, socioeconomic status, locality, and health status were also collected. The online version of the survey was distributed via Qualtrics.

### Analysis

Qualitative content analysis and codebook reliability thematic analyses were employed to analyse the free-text responses related to COVID-19 impact and how technology supported wellbeing, respectively, using an inductive approach [34]. In general, the analyses began by reading participants’ responses to become familiar with the data. This step was followed by open-ended coding guided by the content of the open-ended responses. Initial categories/themes were identified by grouping codes with similar meaning and creating a hierarchical structure of first and second order categories/themes. In line with established approaches and frameworks [35–37], themes/categories were conceptualised as domain summaries derived from the participants’ responses. These domains drove the coding process and constituted the output of the analysis. Through a collaborative and iterative process, categories/themes were reviewed and defined. To ensure the reliability of coding, the identification of codes, categories and themes was conducted with a focus on agreement between multiple coders. The analyses were guided by comprehensive codebooks/coding frameworks, encompassing a list of codes/categories/themes with labels, definitions, instructions on identification, descriptions of any exclusions or qualifications, and illustrative data examples. Using a mixed-methods approach, qualitative data were transformed into quantitative data via frequency counts to determine the prevalence of each category/theme [38, 39]. Further details on each analysis is provided below.

#### COVID-19 impact

Employing a content analysis approach, [40, 41] two researchers (JS and KL) initially analysed a random 5% of responses. This approach was chosen as this method offers a structured and systematic method for organising, categorising, and deriving meaningful insights from large volumes of qualitative data, particularly for national surveys [29, 42–45]. JS and KL familiarised themselves with the data by reading and re-reading participants’ responses. Notes were made of any potential codes by identifying recurring words or units of meaning. During a process of constant comparison, the two researchers worked together to compare, contrast, and consolidate codes into main categories (first order) and categories (second order), resulting in a preliminary coding framework. The two researchers applied the framework to a blinded 27% randomly generated sample of responses to establish inter-rater reliability (kappa = 0.86), per prior studies [43]. The researchers discussed any discrepancies in coding, refined the framework and agreed on final coding decisions. KL then applied the framework to all remaining responses.

Responses were first coded as (1) positive; (2) negative; (3) mixed, that is, contained both positive and negative comments (e.g., “*social isolation is a negative impact as I live alone, however I have kept myself well occupied and have had more time to read and garden and cull possessions no longer needed*”); or (4) ambiguous, i.e., responses were those that did not answer the survey question, or where it was unclear whether the response was positive, negative, mixed or neutral (e.g., “*Stress of 17 yr old son completing yr 12*”, and “*Working from home*”, respectively). Responses which were neutral or ambiguous were not included in further analysis due to the small sample size. Two main categories were developed: Positive Impacts and Negative Impacts. Positive and negative comments, including those from responses classified as ‘mixed’, were further coded under the following second-order categories: Environmental, Physical Health, Social, Mental Health, and Personal. Data analysis thus included ten categories in total (e.g., negative impact – physical; positive impact – physical, etc.) and covered various aspects related to each category (e.g., social participation, social capital, networks were allocated to the category ‘social’).

Responses were assigned to as many categories as were appropriate to cover content, for example, the comment “*because I cannot continue group activities and feel isolated*” was coded under the main category ‘negative impact’ and the categories ‘social’ and ‘mental health’. Responses were analysed using a purpose-designed Microsoft Excel V16.51 spreadsheet. Code frequencies were measured to give an indication of the prominence of different categories and main categories, and descriptive statistics were analysed in SPSS V25.

##### How technology supported wellbeing

For this dataset, inductive codebook reliability thematic analysis [37] was carried out using NVivo V12. Similar to the previous analysis, JS and KL familiarised themselves with the data by reading and re-reading a 5% sample of responses. The two reviewers met to discuss the identification of themes and development of an codebook, which was then then applied to a blinded 26% sample of responses to establish inter-rater reliability by comparing the number of references coded into each theme between the two researchers (kappa = 0.89). KL then applied the codebook to the remaining responses. Reponses that just listed technology types (e.g., YouTube, the Internet) were excluded from the framework as it was unclear how technology was used to support wellbeing [46]. The final codebook comprised four themes: Supported Socialisation, Supported Ongoing and New Activities, Avenue for Information Seeking and Being Informed, and Mental Health Support. Qualitative data was transformed into code frequencies to indicate the prominence of different themes [39]. In addition to answering the survey question, these themes also explain how technology supported older adults during COVID-19 restrictions, and therefore this concept will be referred to as “technology support” from this point forth, encompassing both the support of wellbeing and mitigation of negative impacts.

## Results

### COVID-19 impact

**Fig. 1:**
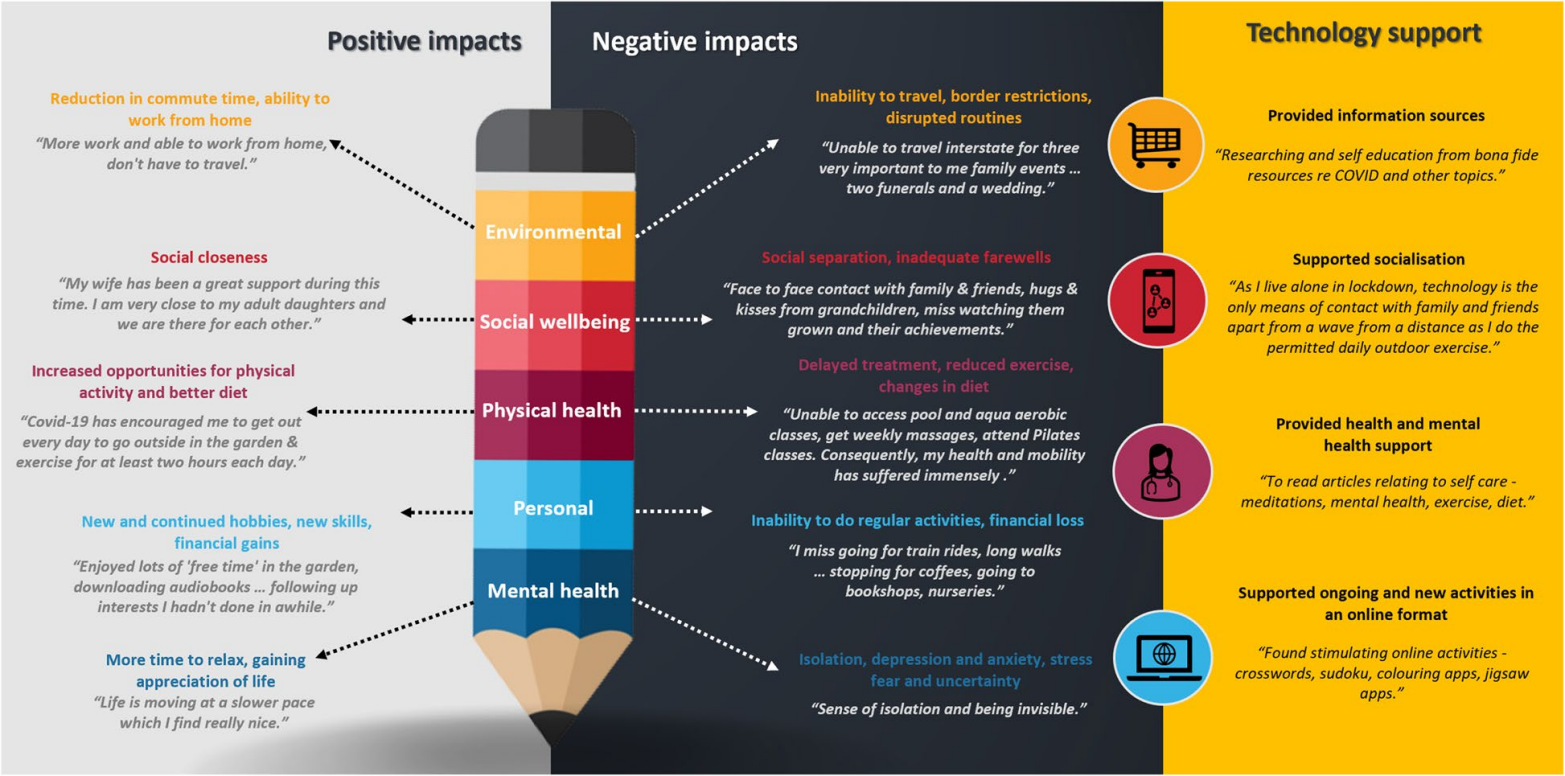
Summary of qualitative categories of COVID-19 impact and themes capturing how technology was able to support wellbeing

Responses were from the online survey only, with no participants opting to complete a paper-based survey. Generally, more negative (*n* = 194, 77.6%) than positive (*n* = 16, 6.4%) and mixed responses (*n* = 40, 16.0%) were provided. A minority of responses were coded as ambiguous (*n* = 2, 0.8%). First-order main categories and second order categories are illustrated with quotes in Fig. 1, with example quotes to provide contextual information. Some of the quotes highlight the inter-relatedness of certain categories, for example, the inability to travel (negative – environmental), was found in some instances to impact access to healthcare (negative – physical health), as well as diminished social connectedness (negative – social), ultimately causing loneliness (negative – mental health).

#### Negative impact

- Negative social impacts, including identification of social isolation, were a common thread in many responses (*n* = 180/191, 94.2%). Older adults were separated from friends and family through national and international border closures, physical distancing measures, and aged care home and hospital restrictions.
*“Husband in aged care and I haven’t been able to see him for 5 weeks. A few FaceTime links but he can’t use phone … he is locked down in his room … no exercise and little contact … very depressing but I can’t look after him at home 24/7 .... need to know when this will end, as his physical and mental health [are] really going down.” (P1522)*

Older adults were unable to participate in social groups such as choirs, exercise groups and sporting groups and missed the interactions they used to have. People also reported cancelling major social events including birthdays and funerals. These negative social impacts were often related to the category of negative impacts on mental health.

*“Reintroduction of Stage 3 & Stage 4 restrictions in Melbourne has reduced my opportunities to interact with other people. Result is profound and soul-destroying loneliness (I live alone).” (P1544)*

While technology supported communication with family and friends, older adults reported missing the connections from face-to-face connections (*n* = 5/191; 2.6%).

*“This winter has been very cold and wet, so even to go outside in the garden has been restricted so the self-isolation on my own is a testing time. It becomes more difficult to stay positive as the weeks go by, phone calls and the computer help but I very much miss face to face contact.” (P985)*

*“I am unable to hug or be tactile to loved ones outside my household.” (P121)*

*“Miss the talking, shaking hands, hugging, overall closeness. Only positive is meeting up on Zoom but it's not the same.” (P1277)*

Some participants (*n* = 3/191; 1.6%) communicated the struggles they experienced not being able to say goodbye to dying loved ones, or not being there to support loved ones through medical treatment.

*“I have just found out my brother’s partner has repaid [rapid] terminal cancer and I won’t get to see her, go to the funeral and support my brother, this is a real tragedy.” (P2500)*

*“Not able to visit/help/connect with dying partner.” (P2439)*

(b) Negative environmental impacts, reported by a third of respondents (*n* = 57/191, 29.8%), were those relating to government restrictions (e.g., mask wearing, not being able to visit cafes or restaurants, stay at home orders), inability to travel, and disruptions to routine activities (e.g., shopping).
  *“Even doing the food shopping etc. you have to social distance and wearing our masks you can’t even share a smile.” (P1573)*
*“My general routines have been completely disrupted. I have been unable to establish a "new COVID routine". Some days I am fine, on other days I have trouble doing anything in my normal routine.” (P505)*

National and international border closures were a frequently reported source of negative environmental impact. For some, this signalled an inability to visit family or friends, and for others, it meant missing out on planned holidays and future travel. For some older adults, working from home was viewed as a negative change to daily routine.

*“Having to accept that, due to isolation and travel restrictions, I may never see my daughter and grandchildren again as they live interstate.” (P2435)*

*“I was on a cruise to England, and it was cancelled after 2 weeks, and I ended up in quarantine in Perth. That wasn’t too bad but getting back was an issue. I feel my chances of doing a trip like that again aren’t good.” (P1412)*

(d) Negative personal impacts were reported by a quarter of the sample (*n* = 65/191, 34.0%) and related to an inability to continue hobbies or participate in spiritual activities such as attending church, as well as loss of finances and/or work. These personal impacts often had cascading, interactive effects on subsequent impacts including negative emotional mental health impacts. Older adults shared their experiences of financial loss, work reductions and restrictions on the ability to engage in volunteer roles.
  *“I am unable to continue my volunteer community work during this period.” (P571)*
*“As self-funded retirees our income is very low and we cannot afford most things. it all gets too much.” (P1295)*

Survey respondents further shared examples of activities, entertainment, and hobbies they could no longer participate in due to lockdowns.

*“Inability to be involved in activities I would normally enjoy...cinemas, restaurants, entertaining at home, live music, U3A (University of the third age), volunteering.” (P1118)*

(iii) Nearly a fifth of the sample reported negative impacts on mental health (*n* = 49/191, 25.7%), which included feelings of depression, anxiety, and stress, including for individuals identified as carers.
*“I have a husband with dementia and Parkinson’s. All his structured activities have ceased. Cognitively he has declined rapidly which has increased my stress levels enormously.” (P2277)*

A commonly raised mental health impact revolved around feelings of acute isolation (*n* = 37/191, 19.4%), that were experienced in waves. For some, isolation was felt despite having some level of existing social contact.

*“Feeling cut off, at times alone. Find my feelings on some days are high and low, never know how bad I'll be on waking. A few times wake positive and ready to face the day head on, others it's an effort to get out of bed.” (P962)*

*“Even [though] I have contact with my family as we live next door to each other, you still feel isolated.” (P1573)*

Another mental health impact reported (*n* = 12/191, 6.3%) was emotions of fear, uncertainty and worry relating to contracting the virus, loss of finances, loved ones’ health, and the future in general.

*“Isolation, fear about the future, money worries, worried for my children.” (P1570)*

*“A sense of fear, primarily about negative health outcome (death) should I catch the virus due to my age and health status as I am the adoptive parent of a 13-year-old.” (P9)*

(d) A small minority of older adults reported negative physical health impacts (*n* = 29/191, 15.2%), such as restricted healthcare access:
*“Not being able to have my medical problems investigated further due to the lockdown and the dangers in visiting medical environments.” (P602)*
*“I have had difficulty caring for my 90-year-old mother and getting her into health care. The health care is very difficult to access in my region. I am anxious that she is deteriorating further and most of her family will not see her before something happens.” (P1295)*

Additional negative physical impacts (n = 8/191, 4.2%) were a reduction in exercise, changes to diet, and weight gain. These health impacts were often reported to result from restrictions to gym and exercise class access:

*“I participated in masters rowing 3-4 times per week prior to the pandemic. In the current restrictions the rowing clubs are all shut. I have gained weight and lost fitness.” (P764)*

*“Inability to maintain an active lifestyle, e.g., 1 hour water aerobics each day, golf 5 times per week, in effect, total removal of physical activities that assist my continued well-being.” (P2645)*

#### Positive impact

- Positive personal impacts were the most commonly reported positive impact (*n* = 30/191, 15.7%) and related to undertaking new and existing hobbies and skills. Lockdowns created more time for people to learn new skills, pursue new interests, or engage in activities that they enjoyed. It also meant that some people were able to save money as they were unable to spend it on outings and travelling costs were reduced.
  *“On the positive side I have completed several outstanding tasks, learned more computer skills such as Zoom, spent more time cooking and reading, been able to cook a treat for my closest neighbours every weekend.” (P2358)*
*“Forced me to slow down and spend less money, more time at home and to begin to take an interest in my garden again.” (P1428)*
- While COVID-19 had many negative impacts on socialisation, it also created positive social impacts for some people (*n* = 20/191, 10.5%). Older adults reported the pandemic provided an opportunity to feel closer to loved ones, to spend more quality time with family at home, to connect with friends and family through technology.
*“More conversations with people, leading to deeper relationships.” (P1060)*
*“Improved relationship because we spend more time together…  Zoom meeting every week with my sisters.” (P373)*
- Positive mental health impacts were also reported by a minority of respondents (*n* = 18/191, 9.4%) and were largely centred around having more time to relax, having a slower pace of life, feeling less pressured, and gaining a greater appreciation. Several respondents spoke about realising what was important in life and saw COVID-19 lockdowns as a period of reflection.
*“The world has slowed down and priorities have in some ways changed. I have become even more aware that I am a very lucky person and very grateful for many things.” (P2596)*
*“Time for self-care and to think about what is best for me without the pressure of work and 'real life'.” (P749)*
- Positive impacts on physical health were mainly focused on opportunities for physical activity (particularly walking) and healthier eating habits. These impacts were described by a small proportion of the sample (*n* = 10/191, 5.2%).
*“Having invested in a walking frame I have been able to take my dog for runs (his, not mine) in the local park so more time walking and chatting with other dog-walkers (social distancing).” (P1053)*
- A minority of survey respondents reported positive environmental impacts of COVID-19 (*n* = 6/191, 3.1%), which included having the flexibility to work from home, and being able to spend more time at home and in the garden, and positive changes to daily routines (e.g., a reduced commute to work).
*“Benefits of being able to work from home in a lovely country environment.” (P581)*

### Technology support

Four themes related to how technology supported wellbeing were identified data. A summary of these themes is depicted in Fig. 1.

#### Supported socialisation

Technology was mostly used to alleviate isolation that was clearly felt by older adults by providing an alternative form of communication to traditional face-to-face socialisation (*n* = 111/242, 45.9%). Technology was perceived to be valuable due to respondents’ ability to connect with family and friends and keep up-to-date with existing social groups (e.g., Zoom book club).

*“Zoom has kept me in touch with my fellow volunteers and also my grandchildren with whom I have been conducting joint cooking sessions online. My book discussion group is a highlight of the month.” (P1100)*

*“Primarily technology has kept me in touch with friends and family, but also provided a window on the world and lessened my feeling of isolation.” (P600)*

These opportunities were described as permitting older adults to maintain a sense of being in touch with others and having some form of security during these times.

*“Regular calls to and from family. Information via phone to save visit. To maintain a sense of being in touch and security.” (P1412)*

#### Supported ongoing and new activities

Technology was broadly used to assist with existing activities, as well occupying older adults with new hobbies (*n* = 98/242, 40.5%). On the one hand, it allowed older adults to continue to work from home. On the other hand, it kept older adults entertained and physically active, and enabled them to continue with essential tasks such as banking and shopping without leaving their homes.

*“I have played a lot of Bridge online, which is one of my passions and it has been a lifesaver for me.” (P636)*

*“Online shopping to minimise exposure and therefore anxiety.” (P470)*

*“Exercise tracking, diet management, searching recipes, attending online fitness classes.” (P1666)*

#### Avenue for information seeking and being informed

Sourcing reliable information about the pandemic was discussed by over a quarter of the sample (*n* = 39/242, 16.1%). Respondents described accessing information related to regulations and COVID-19 updates through online content and social media.

*“Accessing content online to relax, inform and learn about the world - staying up to date with latest developments and stories related to the spread of the pandemic.” (P1194).*

“*Use of podcasts & internet research to get information which is reassuring. From government sites on the internet & Facebook”. (P261)*

Technology was also used to search health-related information such as symptoms.

*“I have looked up symptoms on how I feel and seen what advice they give.” (P1315)*

#### Provision of health and mental health support

Technology was used to assist older adults to keep informed about their health and to facilitate continual communication with healthcare professionals (e.g., telehealth, Zoom counselling). However, this was reported by a minority of the sample (*n* = 16/242, 6.6%). Technology was also used to support mental wellbeing through online material (e.g., reading articles) or digital apps (e.g., for meditation).

*“Yes telehealth. Took pics of my arm; emailed them to doc, telehealth appt.” (P9)*

*“To read articles relating to self-care - meditations, mental health, exercise, diet.” (P276)*

*“I have been using Headspace for mindfulness. Viewing Depression Project app.” (P1757)*

## Discussion

This study reports the experiences of older adults during second lockdowns and how technology was used to support wellbeing. Whilst some respondents reported positive effects of lockdowns, older adults primarily recounted more negative experiences than positive and mixed (positive and negative) responses. Our findings identified the use of technology in assisting socialisation, supporting new and pre-existing activities, providing information, and facilitating access to services in a restricted environment.

COVID-19 restrictions had far-reaching impacts across all domains of life. A rapid review of original studies published on the impact of COVID-19 on older adults extrapolated similar mixed effects of the pandemic on personal, environmental and overall lifestyle including financial situations (e.g., buying more food or water and going out less frequently); health status and behaviour changes (e.g., sleep habits, socialisation, dietary intake, physical activity, alcohol consumption); and electronic product use and social media engagement [47]. Consensus within the literature is that older adults experienced predominantly negative impacts from the pandemic, but to a lesser extent than their younger counterparts [47]. However, it is difficult to elucidate the diverse impacts of COVID-19 whilst accounting for levels of vulnerability amongst older adults and geographical variations in isolation and protective measures implemented between and within countries [47]. 

In Australia, quantitative [14, 25, 48, 49] and qualitative [50–53] studies have investigated the impact of pandemic consequences on older adults (e.g., vaccination acceptance [52], telehealth engagement [49], aged care [25] and elder abuse [51]), however, they have not yet explored with open-ended survey questions the impacts of a second lockdown on older adults in a state with the longest lockdown period at the time. Prior studies tended to provide an overview of immediate impacts from one state [54] or on multistates but on different topics, such as optimism [55], social capital and wellbeing for adults [56] or for individuals with dementia [57]. 

Our findings reflect the major social loss experienced by older adults. Government mandates and disruptions to daily routines led to severe feelings of isolation and a lack of physical contact with participants’ networks. Although technology uptake, social media and software applications were used to reduce loneliness and support mental health during the pandemic [14, 58], older adults also expressed great losses of physical connection and human touch, something technology cannot currently replace.

Online technologies were mostly perceived as favourable, by facilitating social support, sustaining daily activities including hobbies and work, enabling access to essential needs such as banking and shopping, and maintaining health through telehealth services. However, due to our online recruitment strategy our sample was likely to be highly digitally literate, with none completing the postal survey. Regardless, our results indicate that having multiple digital alternatives to support regular social activities were invaluable. Respondents reported the benefits of using teleconferencing services (e.g., Zoom) to engage with previous social groups [59], using software applications (e.g., digital apps) to provide virtual substitutes to fitness and health [60], and telehealth/online content for receiving medical services and improve connections with the external environment [61]. In line with earlier research, digital technologies used for communication can encourage perceived social support from existing and new networks, lower feelings of loneliness, and provide hope during the pandemic [61, 62].

Internationally, positive consequences of COVID-19 amongst older adults are not uniform. For example, adults over 65 in China and Korea generally reported fewer positive effects of the pandemic, however, in the UK the oldest age group reported the most positive impacts (out of six countries) [63]. Although minimal, that international study identified that common benefits centred around more free time and family time [63]. Parallels exist within our study such as the introduction of flexible work from home routines allowing for more idle time; as well as physical, social, and mental health benefits of lockdowns, including the ability to prioritise movement and exercise, foster deeper connections with family and friends, and develop a sense of relief from the pressure of work, finance management and old routines. However, unlike international findings, positive environmental impacts such as reduction in air and noise pollution were not mentioned [63]. Rather, our findings reflect that of previous research amongst the Australian population which highlighted time to connect with family, flexible work arrangements, and a more relaxed lifestyle as the most common explanations for having a positive view towards the COVID-19 restrictions [64].

Our study uniquely found that the most prevalent positive impacts of COVID-19 recurrent lockdowns focused on personal and mental health development opportunities including engaging in joyful activities, acquiring new skills, expressing gratitude, and participating in self-care. This finding could be partially explained by the literature on adaptive emotion regulation strategies which are mechanisms one adopts to cope with difficult life events (e.g., putting into perspective, acceptance and positive refocusing) [65]. Evidence from a large European online survey (*n*= 589) during COVID-19 found that such strategies and behaviours acted as mediators for social support, fear of contracting the virus, loneliness and mental health [66]; whilst another multi-country study (*n*= 1,082) found that engaging in self-care partially mediated the impact of stress on wellbeing during the pandemic [67]. Our findings suggest that although the prevalence of positive impacts were infrequent, it remains possible and important to engineer and encourage behaviours [68] (e.g., via online technology [14]) that produce positive personal and mental health impacts, especially during extended isolation periods to support older adults’ wellbeing in future lockdowns.

### Implications

Unmet needs of an ageing population, as well as the design of services and solutions that fit what older people want and need, have become urgent public health priorities in periods of lockdown [69]. To address and support the health and wellbeing of older adults in future pandemics, governments should adopt an interdisciplinary approach to endorse the value of technology for older adults (i.e., encourage the use of digital solutions for older adults and aim to remove the barriers of digital illiteracy) [61, 69].

Firstly, training older adults to use technology efficiently can be conducted through community group partnerships [20]. Such training should be developed targeting multiple subgroups, including older adults accessing aged care services as well as individuals with low socioeconomic backgrounds and from remote locations, and focus on enabling technology access in addition to developing essential skills to access online services (e.g., grocery stores, food delivery, telehealth consultations) and social networking skills [20].

With an ageing population, it is paramount that technology remains cost-friendly to mitigate unaffordability, particularly given the effects of socioeconomic status found on negative impacts. Moreover, communities should adopt the feedback of older adults as criteria to tailor resources and tools that can assist them in feeling more confident in their use of technology or navigation through websites/social media platforms [61]. Measures to promote the accessibility of resources could also prove to be helpful during periods of social isolation. Overall, it is imperative to maintain and expand on the positive benefits of pre-existing strategies used to improve the health and wellbeing of older populations in future lockdowns.

### Strengths and limitations

A strength of our study was the systematic methodology used to qualitatively appraise the large data source. Although qualitative analysis inherently involves subjectivity (i.e., coder is required to make decisions about coding and themes), analyses were conducted independently by two researchers who followed a transparent procedure to reduce partiality, guided by the development of a codebook. Further, inter-rater reliability was established for blinded samples in each analysis.

Our study has several limitations. First, the results that we present are primarily descriptive in nature is restricted to a relatively small sample size, and we are unable to establish a causal relationship between technology use and wellbeing. Similarly, the mean age of the sample was relatively young (67 years) and thus may not reflect the experience of much older adults. As the sample included adults of working age, they may be more likely to have access to and be familiar with technology programs through workplaces. As such, the positive benefits of technology in supporting wellbeing may be ungeneralisable to those with less access and confidence in using technology programs for communication. Second, there may also be non-response biases as well as an inherent risk of bias in free-text responses towards respondents who are more literate, have English as a first language, who do not have learning difficulties and those who have adequate technology literacy*.* Third, existing differences between respondents who left a free-text comment and those who did not could reflect a skewed perspective. Future, more inclusive, larger sample studies are required to establish a thorough understanding of impact over time and mitigate factors to support the personal, social, physical, emotional and environmental impacts resulting from COVID-19 restrictions. Additionally, data from other sources or methods were not incorporated, which may have provided a more comprehensive understanding of the impacts. While our study included free-text questions aimed at providing context-specific insights, these questions lacked a multi-dimensional approach (e.g., phrasing of either negative or positive impact). Thus a broader methodological approach could enhance the study's robustness and triangulate findings for a more holistic interpretation. Future research may consider integrating qualitative interviews, observations, or complementary data sources to address the potential limitations associated with relying solely on the responses to two open-ended questions in the survey.

## Conclusion

Findings from this study offer a better understanding of how older Australians experienced second lockdowns, highlighting the prevailing repercussions of COVID lockdowns and accentuating the value of technology in supporting connectivity. The results indicate the importance of addressing personal, social, physical, environmental and emotional consequences of COVID-19 restrictions for older adults, and the provision of adequate access and literacy surrounding technology resources targeting subgroups may be particularly critical to crisis management.

## Data Availability

The aggregate data that support the findings of this study are available from request from the primary author ( https://www.joyce.siette@westernsydney.edu.au).
